# Upadacitinib is effective in treating psoriasis combined with lichen planus: a case report

**DOI:** 10.3389/fmed.2026.1771710

**Published:** 2026-04-20

**Authors:** Xingmo Li, Jinxu Qi, Liping Shi, Lijuan Liu, Xiaoyu Xie, Guoqiang Zhang, Qing Zhu

**Affiliations:** 1 Department of Dermatology, The First Hospital of Hebei Medical University, Shijiazhuang, Hebei, China; 2 Subcenter of National Clinical Research Center for Skin and Immune Diseases, Shijiazhuang, Hebei, China; 3 Hebei Provincial Innovation Center of Dermatology and Medical Cosmetology Technology, Shijiazhuang, Hebei, China

**Keywords:** comorbidity, lichen planus, psoriasis, treatment, upadacitinib

## Abstract

Psoriasis frequently coexists with other immune-mediated conditions such as arthritis, alopecia areata, vitiligo, and hidradenitis suppurativa, many of which remain challenging to manage. To date, there have been rarely reported cases of treating psoriasis combined with lichen planus. We report a case of coexisting psoriasis and lichen planus in a female patient. Despite initial efficacy with acitretin and topical mometasone, the patient self-discontinued treatment over safety concerns, prompting a relapse. Upadacitinib was subsequently initiated and yielded marked improvement within 3 months. By discussing the disease interplay and treatment rationale, this report seeks to inform clinical decision-making.

## Highlights

- In this work, we reported a case of psoriasis combined with lichen planus for the first time and our experience about using upadacitinib for treatment, we also discussed the possible pathogenic mechanisms.
- We hope our research can fill the gap in the field of psoriasis comorbidities and provide treatment experience.

## Introduction

Psoriasis is a chronic inflammatory skin disease characterized by excessive proliferation and abnormal differentiation of keratinocytes, typically presenting with erythematous lesions, impacting the patient’s quality of life (1). It is frequently associated with various immune-mediated comorbidities, including psoriatic arthritis, inflammatory bowel disease, alopecia areata, vitiligo, and hidradenitis suppurativa, and the related mechanisms may involve the interactions of immune cells (Th17 cells and lymphocytes) and inflammatory factors (IL-17A) (2–6), and the biologic treatment of psoriasis may affect the progression of comorbidities (4). However, there are few reports of psoriasis combined with lichen planus. Our article reported a rare case of the coexistence of two diseases.

Lichen planus is a chronic, idiopathic inflammatory disease primarily caused by T-cell mediated immune response, affecting the skin, mucous membranes (especially the oral cavity) and appendages. Treatment remains challenging due to its chronic and often refractory nature (7). Since both psoriasis and lichen planus involve T cells, we infer that the occurrence of lichen planus after psoriasis may be related to a persistent dysregulation of the immune system. After treatment or remission of psoriasis, the T cell response may redirect toward other targets, such as the basal layer keratinocytes, thereby triggering a pathological process similar to lichen planus (8). Upadacitinib is an oral highly selective small molecule JAK-1 inhibitor. Compared to other JAK inhibitors, it has better efficacy and safety (9). JAK-mediated signaling pathways are involved in various immune-related dermatological diseases (10). As of October 2025, the FDA-approved indications for upadacitinib include: Adults with moderate to severe rheumatoid arthritis when 1 or more medicines called tumor necrosis factor (TNF) blockers have been used, and did not work well or could not be tolerated. Adults with active psoriatic arthritis when 1 or more medicines called TNF blockers have been used, and did not work well or could not be tolerated. Adults with active ankylosing spondylitis when 1 or more medicines called TNF blockers have been used, and did not work well or could not be tolerated. Adults with active non-radiographic axial spondyloarthritis with objective signs of inflammation when a TNF blocker medicine has been used, and did not work well or could not be tolerated. Adults with giant cell arteritis. Adults with moderate to severe ulcerative colitis when 1 or more medicines called TNF blockers have been used, and did not work well or could not be tolerated, or after taking a different injection or pill (systemic therapy) when your healthcare provider does not recommend TNF blockers. Adults with moderate to severe Crohn’s disease when 1 or more medicines called TNF blockers have been used, and did not work well or could not be tolerated, or after taking a different injection or pill (systemic therapy) when your healthcare provider does not recommend TNF blockers (11). Here we reported a case of a female patient with psoriasis and lichen planus, and we attempted to use upadacitinib to treat her; after 3 months of treatment, we achieved good efficacy.

## Case report

The patient was a 35-year-old female, with a height of 1.65 m and a weight of 70 kg. She presented to our outpatient clinic in May 2025 with the chief complaint of “erythematous plaques with pruritus on the lower back for 3 years, and newly developed erythematous plaques and papules on the extremities with pruritus for 1 year.” Three years prior, several palm-sized erythematous plaques with scaling had developed on her lower back without any obvious precipitating factors, accompanied by pruritus that led to unconscious scratching. The patient had previously visited a local hospital, where psoriasis was diagnosed on the basis of dermatoscopic examination and other assessments; however, the severity of her skin lesions at that time was difficult to retrospectively determine. She was prescribed oral Xiaoyin Granules and Compound Glycyrrhizin Tablets, as well as topical Halometasone Cream and Calcipotriol Ointment, resulting in partial improvement of the skin lesions. She did not attend regular follow-up visits thereafter. One year ago, new erythematous plaques and papules developed on her extremities without obvious cause, also accompanied by pruritus. She attempted treatment with levocetirizine, which proved ineffective. She subsequently revisited the local hospital, where she was prescribed oral acitretin and topical Mometasone Furoate Cream. Due to concerns regarding potential severe adverse effects associated with long-term acitretin use, the patient self-discontinued the medication and was ultimately referred to our department for further treatment. The patient’s medical history was unremarkable for chronic diseases, malignancies, tuberculosis, hepatitis, or thrombosis. However, there was a positive family history of psoriasis, as her mother had been affected for over 20 years.

**FIGURE 1:**
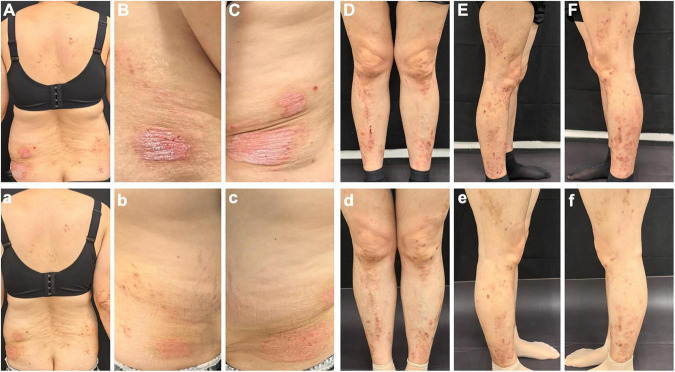
Before treatment, erythema and papules can be seen on the lower back **(A–C)**, and limbs **(D–F)**, with rough surfaces, scratch marks and blood scabs visible; after 3 months of upadacitinib, erythema and papules on the lower back **(a–c)** and limbs **(d–f)** reduced.

**FIGURE 2:**
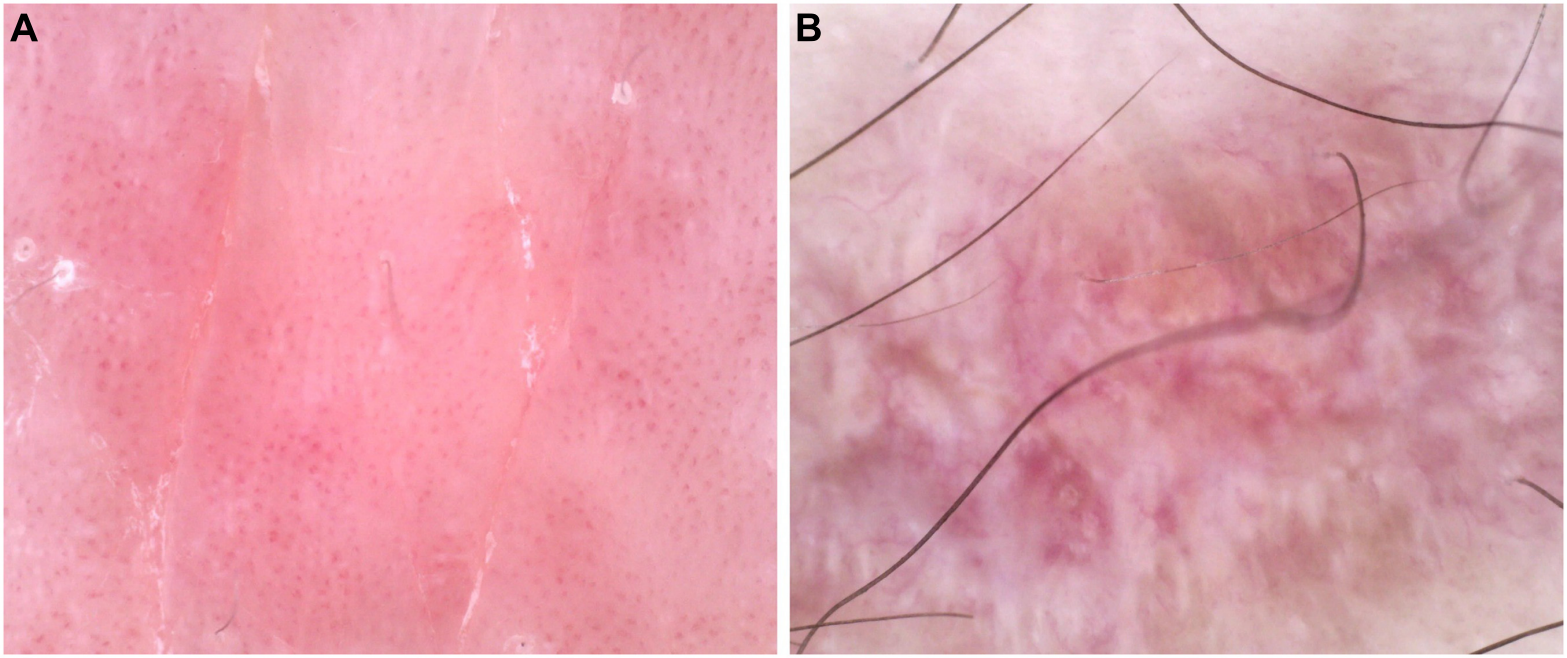
**(A)** The lower back dermoscopy revealed a bright red background with dotted, small spherical shapes, hairpin and ring blood vessels, and scattered white scales, considering psoriasis. **(B)** The lower leg dermoscopy revealed prominent white reticular streaks (Wickham striae) and a mixed appearance of radially arranged linear blood vessels, considering lichen planus.

**FIGURE 3:**
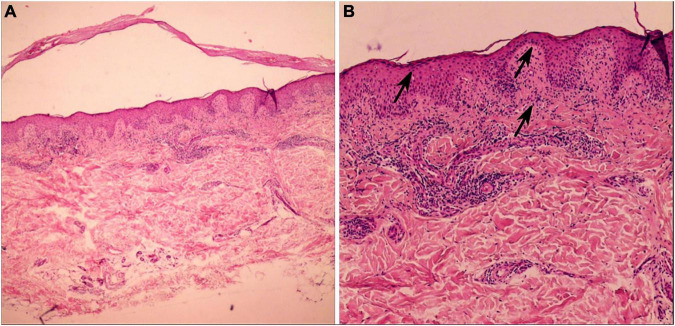
The lower leg histopathology showed hyperkeratosis of the epidermis, wedge-shaped thickening of the granular layer, irregular hyperplasia of the spinous layer, liquefaction and degeneration of basal cells, visible pigment incontinence, and roughly band-like infiltration of lymphocytes in the superficial dermis. **(A)** H&E, × 40; **(B)** H&E, × 100).

Dermatological examination revealed erythematous, thickened plaques with silvery-white scales on the lower back, with auspitz sign, candle wax sign, and membrane phenomenon. The involved Body Surface Area (BSA) was 1.8%. Light red lichen-like plaques can be seen on the limbs, with a rough surface and scattered polygonal flat papules at the edge. Nails, joints and hair were not affected (Figure 1). Dermoscopy of the lower back showed a bright red background with regularly distributed dotted vessels with white scaling, findings suggestive of psoriasis. Dermoscopy of the lower limbs revealed distinct white Wickham’s striae and linearly arranged, radially distributing vessels, suggestive of lichen planus. A skin biopsy was recommended for histopathological confirmation (Figure 2). Laboratory tests and imaging examinations were conducted, including complete blood count, urinalysis, liver and renal function, lipid profile, blood glucose, electrolytes, coagulation profile, tumor markers, antibodies for hepatitis B and C, interferon-gamma release assay (IGRA), and chest computed tomography (CT) scan, and they showed no abnormalities. We recommended separate histopathological biopsies for the lesions on the lower back and the lower limbs. Unfortunately, the patient was strongly averse to any invasive procedures. After thorough discussion, she consented only to a biopsy of the lower limb lesion (Figure 3). The patient’s skin lesions developed in a clear sequential order. Lichen planus-like lesions appeared 2 years later than the psoriasis-like lesions, and the characteristics of the lesions at the two sites were distinct. Further dermoscopic examination revealed that the lower back lesions exhibited typical Auspitz sign, candle wax sign, and membrane phenomenon, while the lower limb lesions showed typical Wickham’s striae. These features did not co-occur within a single lesion. The histopathological diagnosis of the lower limb lesions was lichen planus, with no indication of psoriasis overlap. Based on the above analysis, we consider the possibility of psoriasiform lichenoid overlap to be low.

Considering that the patient had symptomatic lichen planus, a history of previous medication, and concerned about adverse reactions after taking the medicine, we ultimately chose upadacitinib at a dose of 15 mg once a day, which was an off-label medication. After 3 months of therapy, the erythematous plaques on the lower back and extremities showed marked improvement (Figure 1). Her Physician’s Global Assessment (PGA) score decreased from 3 to 1, the Visual Analog Scale (VAS) score decreased from 6 to 2, and both the Psoriasis Area and Severity Index (PASI) and Dermatology Life Quality Index (DLQI) scores improved from 2.1 to 0.9 and from 10 to 3, respectively. The patient also reported substantial reduction in pruritus, with improved social engagement and greater willingness to appear in public without concern regarding her appearance. Although there has been an improvement in clinical practice, the lack of a formal severity score for lichen planus may have limited the efficacy of ulapatinib in treating lichen planus. No adverse events were observed during treatment. Laboratory investigations, including complete blood count, urinalysis, comprehensive biochemistry, interferon-gamma release assay (IGRA), and hepatitis B serology, revealed no significant abnormalities, confirming a favorable safety profile and supporting treatment continuation. The patient subsequently opted for continued follow-up at a local hospital. A recent telephone follow-up confirmed that her condition continues to improve. Although laboratory assessments and telephone follow-ups were conducted, the duration of follow-up remains limited. This constraint affects our ability to fully evaluate the long-term efficacy and safety profile of JAK inhibitor therapy. We will continue to monitor and follow up on the patient’s condition and laboratory tests in the future.

## Discussion

Psoriasis is a T-cell-mediated autoimmune disease characterized by abnormal keratinocyte proliferation and differentiation, along with inflammatory cell infiltration. Its pathogenesis involves various cytokines (such as IL-17, IL-23, TNF-α, IFN-γ) and signaling pathways (such as the JAK-STAT pathway) (12). Emerging evidence indicates that dendritic cells, Th17 cells, and keratinocytes form a pathogenic triad in psoriasis. Dendritic cells secrete TNF-α and IL-23, driving the differentiation of T cells into Th17 cells, which in turn produce pivotal cytokines like IL-17, IFN-γ, and IL-22. The signaling of these cytokines is mediated, to varying degrees, by the JAK-STAT pathway (13). In the lesional skin of lichen planus, dendritic cells are characteristically clustered in a lichenoid band-like infiltrate at the dermal-epidermal junction, where they closely interact with T cells and plasma cells, constituting a key component of the classic inflammatory infiltrate (14). In addition, it is also affected by genetic susceptibility and environmental factors such as infection and stress, leading to excessive activation of immune cells and angiogenesis (15). The main effector cells in lichen planus are CD8^+^ T cells, they participate in cytotoxic mechanisms by triggering keratinocyte apoptosis through granule exocytosis mediated by Fas-FasL and TNF-α receptor interactions, resulting in epidermal damage (16). Additionally, a study had shown that IFN-γ is significantly upregulated in the oral mucosa and skin lesions of patients with lichen planus (17). Thus, psoriasis and lichen planus share important pathogenic features: both are T-cell-mediated autoimmune conditions in which IFN-γ serves as a key driver, resulting in keratinocyte dysfunction and destruction. Both diseases also involve the JAK-STAT pathway, highlighting its potential as a common therapeutic target. In addition, epigenetic regulation and oxidative stress are believed to contribute to the pathophysiology of both disorders (18, 19).

The development of lichen planus following psoriasis in our patient may be explained by several interconnected mechanisms. First, sustained psoriatic inflammation might contribute to the activation of additional T-cell subsets, such as CD8^+^ T cells, a process that could potentially underlie the development of a lichen planus-like pathology. Second, the co-occurrence of the two conditions may involve shared inflammatory mediators within the local tissue milieu; for example, IFN-γ produced in psoriatic lesions could participate in activating immune pathways that are also implicated in lichen planus. Furthermore, available evidence suggests associations between lichen planus and alterations in natural killer (NK) cell function, metabolic pathways (including those involving picolinate and nicotinic acid metabolites), and the presence of senescent mesenchymal cells. These associated factors are hypothesized to contribute to a dysregulated local immune environment, which may help to perpetuate inflammation. It is plausible that the chronic inflammatory state in psoriasis similarly alters the cutaneous metabolic milieu and promotes the accumulation of such senescent cells, thereby facilitating the onset of lichen planus (20, 21).

This case highlights the potential of upadacitinib, a selective JAK1 inhibitor, in managing coexisting psoriasis and lichen planus—a clinical scenario for which no standard treatment currently exists. As previously discussed, the shared involvement of the JAK-STAT pathway in both diseases provides a rational basis for JAK inhibitor therapy. JAK-mediated signaling is implicated in a broad spectrum of immune-mediated dermatoses, including atopic dermatitis (via IL-4, IL-5, IL-13), psoriasis (via IL-17, IL-23), dermatomyositis (via IFN-α, TNF-β, IL-6, IL-15), and vitiligo (via IFN-γ), among others (10). Supporting this approach, a network meta-analysis demonstrated that JAK inhibitors are superior to placebo in achieving PASI 75 response in psoriasis patients at both 8 and 12 weeks (22). Furthermore, a systematic review reported that 73.3% of lichen planus patients achieved partial or complete remission following JAK inhibitor treatment (23). Studies have revealed that the receptors for IFN-γ, IL-21, IL-6, and type I interferons—cytokines found to be elevated in lichen planus—all share a common reliance on JAK1 for signal transduction, ultimately leading to the activation of STAT1 (17), further corroborating their efficacy in this condition.

Upadacitinib is a highly selective JAK1 inhibitor that had been approved for the treatment of inflammatory diseases such as atopic dermatitis, rheumatoid arthritis, psoriatic arthritis, ulcerative colitis, and ankylosing spondylitis. It can partially block the JAK-STAT signaling pathway located downstream of cytokines, inhibiting the expression of key inflammatory cytokines such as IFN-γ, thereby blocking the expression of downstream inflammatory mediators, resulting in disease remission (17, 24). In addition, upadacitinib can reduce the excessive proliferation of keratinocytes by downregulating the expression of IL-22 and promote the synthesis of skin barrier proteins such as filaggrin, restoring epidermal barrier function, it also can quickly alleviate itching in atopic dermatitis (25). The high selectivity of upadacitinib can reduce the inhibition of JAK2 and the risk of anemia caused by interference with erythropoietin signaling mediated by JAK2 (26), and it has shown an acceptable safety profile in a real-life study (27). Balestri et al. described a 45-year-old female patient with lichen planus and peripheral psoriatic arthritis. She showed notable improvement in oral lichen planus lesions after 1 week of treatment with upadacitinib (15 mg once a day), and the improvement was sustained at the 12-week follow-up (28). Koohbaran et al. reported a 59-year-old female patient with lichen planus, in whom oral lesions completely resolved following 24 weeks of treatment with upadacitinib (15 mg once a day) (29). A review of the literature summarizing the off-label applications of upadacitinib in dermatology, based on a compilation of previous case reports, suggests that upadacitinib has demonstrated limited efficacy in treating a variety of conditions. These include lichen planus, psoriasis, pyoderma gangrenosum, Sweet’s syndrome, hidradenitis suppurativa, discoid lupus erythematosus, chronic actinic dermatitis, and bullous pemphigoid. These findings indicate that upadacitinib possesses significant clinical potential beyond its currently approved therapeutic indications (30).

This case report was limited by its single-case nature, relatively short follow-up duration, and the lack of a formal scoring system to quantify the severity of lichen planus.

## Conclusion

Our study suggests the potential therapeutic utility of upadacitinib in the management of coexisting psoriasis and lichen planus. However, the findings are limited by the study design as a single case report and a relatively short follow-up duration. Future investigations involving larger case series and controlled clinical trials are warranted to validate the efficacy of upadacitinib for this specific comorbidity and to establish standardized treatment protocols.

## Data Availability

The original contributions presented in this study are included in this article/supplementary materials, further inquiries can be directed to the corresponding authors.
